# Toward learning Lattice Boltzmann collision operators

**DOI:** 10.1140/epje/s10189-023-00267-w

**Published:** 2023-03-06

**Authors:** Alessandro Corbetta, Alessandro Gabbana, Vitaliy Gyrya, Daniel Livescu, Joost Prins, Federico Toschi

**Affiliations:** 1 Eindhoven University of Technology, 5600 Eindhoven, MB The Netherlands; 2 Los Alamos National Laboratory, Los Alamos, NM 87545 USA; 3 Consiglio Nazionale della Ricerche-IAC, Rome, Italy

## Abstract

**Abstract:**

In this work, we explore the possibility of learning from data collision operators for the Lattice Boltzmann Method using a deep learning approach. We compare a hierarchy of designs of the neural network (NN) collision operator and evaluate the performance of the resulting LBM method in reproducing time dynamics of several canonical flows. In the current study, as a first attempt to address the learning problem, the data were generated by a single relaxation time BGK operator. We demonstrate that vanilla NN architecture has very limited accuracy. On the other hand, by embedding physical properties, such as conservation laws and symmetries, it is possible to dramatically increase the accuracy by several orders of magnitude and correctly reproduce the short and long time dynamics of standard fluid flows.

**Graphic abstract:**

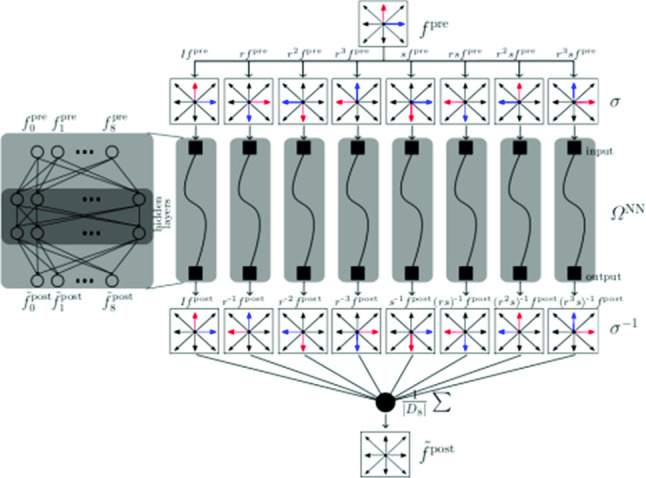

## Introduction

The Lattice Boltzmann Method (LBM) is a computationally efficient method for the simulation of fluid flows in a wide range of regimes. LBM allows solving a set of macroscopic equations via the time evolution of a (minimal) discrete version of the continuum Boltzmann equation, following the stream and collide paradigm.

While its original formulation targets mostly isothermal weakly compressible fluid flows, over the years several algorithmic developments have allowed extending the method to support the simulation of a wide range of complex flows, such as multi-phase [1, 2], turbulence [3], thermo-hydrodynamics [4, 5], non-Newtonian flows [6, 7], radiative transport [8], semi-classical fluids [9], relativistic flows [10], and many others [11], with an outlook toward exa-scale computing [12]. Most of these algorithmic enhancements have targeted the modeling of the collision process and, as a result, a large variety of collision models have been proposed to extend the applicability and overcome the shortcomings of the standard LBM. Notable examples extending the single relaxation time Bhatnagar–Gross–Krook (BGK) collision operator [13] are given by the two relaxation times (TRT) [14], multi-relaxation time (MRT) [15, 16], which can be combined with regularization procedures [17–19], and local viscous corrections, ensuring the validity of the H-theorem after the velocity discretization [20, 21]. More recent developments have taken into consideration the ellipsoidal statistical BGK [22] and the Shakov model [23], which allow to decouple the thermal relaxation from the viscous one. They also made possible to compute equilibrium distributions numerically, in principle, allowing to reproduce an arbitrary number of moments of the Maxwell-Boltzmann distribution [24]. For a comprehensive review comparing collision models for LBM the interested reader is referred to [25].

In recent years, there has been an increased interest in adoption of machine learning (ML) models, typically, of artificial neural networks (NN), to approximate various kernels/operators in the simulation of physical systems. Artificial neural networks form a class of nonlinear parametric models satisfying universal approximation property [26]. This property coupled with efficient computational tools for automatic differentiation and sensitivity analysis of forward and backward propagation, in the last decade, has led to outstanding results in such fields as computer vision [27] and natural language processing [28].

However, until recently, the biggest achievements of ML in scientific environment have been limited to approaches that are data-driven but agnostic to traditional scientific modeling of the underlying physics. Integrating the modern ML with physical modeling is the major challenge of what we call today Physics-Informed Machine Learning (PIML) [29, 30]. In particular, in fluid dynamics, there has been significant PIML activity in recent years. Examples include embedding physical constraints, such as Galilean invariance and rotational invariance, into the closure model [31, 32] and PIML models infusing physical constraints into the neural networks [33, 34]. Other efforts on turbulence modeling are summarized in [35, 36]. In addition to developing closure models, novel ML approaches have been used to learn turbulence dynamics [37], where a Convolutional Long Short Term Memory (ConvLSTM) Neural Network was developed to learn spatial-temporal turbulence dynamics; study super-resolution allowing to reconstruct turbulence fields using under-resolved data [38]; use Neural Ordinary Differential Equation (Neural ODE) for turbulence forecasting [39]; or measure [40], model and control flows [41].

Up to now, very few works have proposed applications of ML to LBM. Most of these have been focusing on accelerating the calculation of steady-state flows using convolutional neural networks [42–44], while Bedrunka et al. [45] employed a fully connected feed-forward neural network to tune the parameters of a MRT collision operator.

Since LBM entails a mesoscopic representation, it employs substantially more degrees of freedom (i.e., the number of discrete particle distribution functions) than the macroscopic observables of interest. These extra degrees of freedom suggest a possibility of using ML to encode more information in the model in order, for example, to extend its applicability, accuracy, and enhance the numerical stability. Indeed, deriving collision operators for LBM that can handle different type of fluid flows is an open problem with a lot of ongoing research, therefore there is a need for new and more general approaches, and data-driven techniques may offer an answer to this quest.

In this work, we take a first step in this direction and consider the problem of learning a custom collision operator from reference data. The collision operator will be represented by a NN that takes as inputs pre-collision and return post-collision populations. As a proof-of-concept we evaluate different neural network architectures to identify design choices that improve performance of the learned collision operator. To make performance evaluation more straightforward, we consider a large synthetic dataset containing pre- and post-collision populations pairs that itself was generated by a collision operator, specifically the BGK collision operator. In theory, in the limit of infinite data and infinite training resources it should be possible to recover the underlying operator. On the other hand, in practice, there will always be an error that (as we show later) significantly depends on the architecture of the NN. We show that constraining the NN to respect physics properties such as conservation laws and symmetries is key for accuracy. We evaluate the accuracy of the learned collision operator on both single-step (static) collision, as well as multi-step (dynamic) collisions, interleaved with advection steps, for the simulation of standard benchmarks. The focus of this work is on exposing the main ingredients needed to accurately learn a collision operator from data, while, for the moment, no attention is paid to computational efficiency.

This article is structured as follows: in Sect. 2, we provide a brief description of the Lattice Boltzmann Method. In Sect. 3, we define a PIML approach for learning a collision operator from data, focusing in particular on the embedding of relevant physical properties. In Sect. 4, we report simulations results for two numerical benchmarks where we have replaced the collision term in LBM simulations with a neural network. Here, we also compare the accuracy achieved by different neural network architectures. Concluding remarks and future directions are summarized in Sect. 5.

## Lattice Boltzmann method

In this section, we give a short introduction to the Lattice Boltzmann Method; the interested reader is referred to, e.g., Ref. [11, 46] for a thorough introduction.

LBM simulates the evolution of macroscopic quantities (such as density and velocity) through a mesoscopic approach based on the synthetic dynamics of a set of discrete velocity distribution functions

$$\begin{aligned} f_i(\varvec{x},t), \ i = 0, \dots , q-1, \end{aligned}$$

to which we will refer as lattice populations.

At each grid node $\varvec{x}$, the lattice populations are defined along the discrete components of the stencil $\{ \xi _i \}, \ i = 1, \ldots , q-1$. It is customary to distinguish between different LBM schemes using the D*d*Q*q* nomenclature, in which *d* refers to the number of spatial dimensions and *q* to the number of discrete components.

**Fig. 1:**
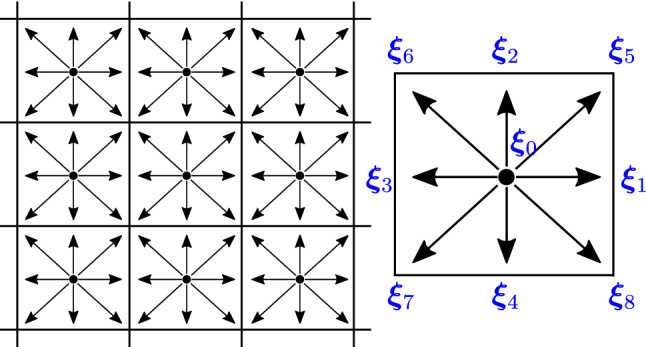
Example of a $3\times 3$ LBM grid (with a single grid point shown on the right hand side) making use of the D2Q9 model where the lattice populations can move along 9 possible directions

In this work we adopt the D2Q9 model, based on the stencil in Fig. 1, where populations can move along 9 possible directions, defined by the following discrete velocity vectors:

$$\begin{aligned} \varvec{\xi }_i= {\left\{ \begin{array}{ll} (0,0) &{} i = 0,\\ (1,0),( 0,1),(-1, 0),(0,-1) &{} i = 1,2,3,4,\\ (1,1),(-1,1),(-1,-1),(1,-1) &{} i = 5,6,7,8. \end{array}\right. } \end{aligned}$$

In general, the velocity sets, $\varvec{\xi }_{i}$, are chosen such that any spatial vector $\varvec{\xi }_{i} \varDelta t$ points from one lattice site to a neighboring lattice site. This guarantees that the populations $f_i$ always reach another lattice site during a time step $\varDelta t$.

The time evolution of each lattice population is ruled by the lattice Boltzmann equation which, in the absence of external forces, reads as:

1 $$\begin{aligned} f_i(\varvec{x}+ \varvec{\xi }_{i} \varDelta t , t + \varDelta t) - f_i(\varvec{x},t) = \varOmega \left( f_i(\varvec{x},t) \right) , \end{aligned}$$

where $\varOmega$ is the collision operator. Among various possible choices, in this work we consider the BGK [13] operator

2 $$\begin{aligned} \varOmega (f_i(\varvec{x},t)) = -\frac{\varDelta t}{\tau } \left( f_i(\varvec{x},t) - {f}_{i}^{\textrm{eq}} (\varvec{x},t)\right) , \end{aligned}$$

which models collisions as a linear relaxation process of the distribution function toward its equilibrium. Here, $\tau$ is the relaxation time, $\varDelta t$ is the time step, and $f_i^{\textrm{eq}}(\varvec{x},t)$ is the discrete equilibrium distribution, for which we employ a second-order Hermite-expansion of the Maxwell-Boltzmann distribution:

3 $$\begin{aligned} f^{\textrm{eq}}_{i}(\rho , \varvec{u}) = \, w_i \rho \left( 1 + \frac{ \varvec{u} \cdot \varvec{\xi }_{i}}{c_s^2} + \frac{(\varvec{u} \cdot \varvec{\xi }_{i})^2 -(c_s |\varvec{u}|)^2 }{2 c_s^4}\right) , \end{aligned}$$

with $w_i$ a lattice-dependent set of weighting factors. For the D2Q9

$$\begin{aligned}{} & {} w_0 = 4/9,\ \ w_1=w_2=w_3=w_4=1/9,\\{} & {} w_5=w_6=w_7=w_8=1/36. \end{aligned}$$

In lattice units, $\varDelta t=1$, while the speed of sound in the lattice for the D2Q9 model is $c_s = 1 / \sqrt{3}$. Finally, $\rho$ and $\varvec{u}$ indicate, respectively, the macroscopic density and the velocity fields. These macroscopic observable can be computed in terms of the moments of the velocity distribution functions as

4 $$\begin{aligned} \rho = \sum _{i=0}^{q-1} f_i \qquad \text {and} \qquad \rho \varvec{u} = \sum _{i=0}^{q-1} f_i \varvec{\xi }_{i}. \end{aligned}$$

Following an asymptotic analysis, like the Chapman-Enskog expansion [47], it can be shown that Eq. 1 delivers a second-order approximation of the Navier-Stokes equations. In particular, the following relation between the relaxation time parameter $\tau$ and the kinematic viscosity $\nu$ of the fluid holds:

5 $$\begin{aligned} \nu = \left( \tau - \frac{1}{2} \right) c_s^2 . \end{aligned}$$

We conclude this section by sketching the LBM algorithm. Provided a suitable initialization of the particle distribution functions, each time iteration of the algorithm entails the following steps:

1. Perform the streaming step:
  6 $$\begin{aligned} f_i^{\textrm{pre}}(\varvec{x}, t) = f_i(\varvec{x} - \varvec{\xi }_{i} \varDelta t , t) . \end{aligned}$$
2. Compute the macroscopic fields using Eq. 4
3. Calculate the equilibrium distribution function using Eq. 3
4. Apply the collision operator
  7 $$\begin{aligned} f_i^{\textrm{post}}&= f_i(\varvec{x}, t + \varDelta t) = f_i^{\textrm{pre}}(\varvec{x},t) \nonumber \\&\quad - \frac{\varDelta t}{\tau } \left( f_i^{\textrm{pre}}(\varvec{x}, t) - {f}_{i}^{\textrm{eq}} ( \rho (\varvec{x},t), \varvec{u}(\varvec{x},t) ) \right) . \end{aligned}$$

### Collision invariants and equivariances

The operator $\varOmega$ carries physical properties of the Boltzmann collision, which can be phrased in terms of invariances and equivariances. Respecting these physical aspects will turn central in the performance of the machine learning models discussed in the next sections. In particular, $\varOmega$ satisfies the following:

**P1**: *Scale equivariance.* Scale factors $\lambda > 0$, remodulating all the pre-collision populations, are preserved, i.e.,
  8 $$\begin{aligned} \varOmega (\lambda f_i^{pre}) = \lambda \varOmega ( f_i^{pre})\ . \end{aligned}$$
  In other terms, the collision is degree-1 homogeneous.
**P2**: *Rotation and reflection equivariance.* Generic two-dimensional collisions are equivariant with respect to the 2-dimensional orthogonal group *O*(2). This translates into the rotational and mirror independence on the spectator viewpoint. As we restrict to a D2Q9 lattice, this property reduces to preserving the 8th-order dihedral symmetry group of the lattice $D_{2n}\subset O(2)$, $n=4$. This group is generated by a 90 degree rotation and a mirroring with respect to symmetry axes of the cell (e.g., the *x* axis). Naming these two operations, respectively, *r* and *s*, and identifying with *I* the identity operation, the 8 elements of $D_{8}$ are
  9 $$\begin{aligned} D_8 = \{I,r,r^2,r^3,s,rs,r^2s,r^3s\}. \end{aligned}$$
  Here, the *n*-th power indicates *n* subsequent applications of the same operator (i.e., $r^2$ is a 180 degree rotation).

In 3-dimensions the extension of the dihedral symmetry group contains 48 elements.

**Fig. 2:**
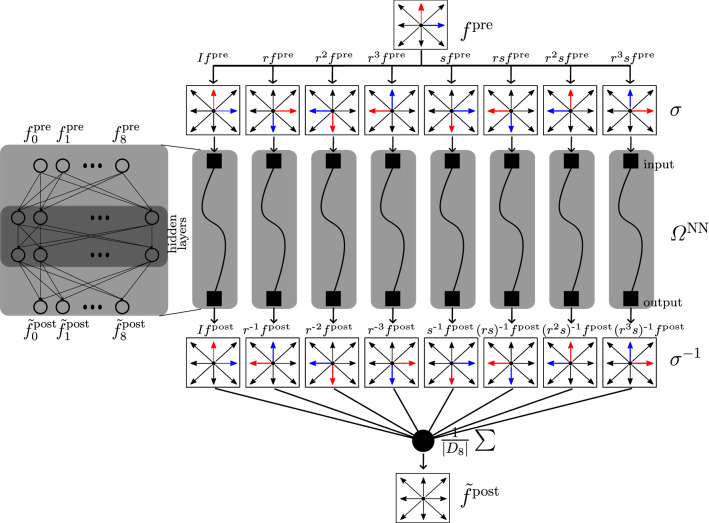
Sketch of a Neural Network architecture implementing the group averaging method. The core network (gray box on the left hand side) is evaluated 8 times on rotated/shifted versions of the input. The inverse transformation is applied to the 8 outputs which are then averaged in order to produce the final prediction

When applied to the populations, these operators effectively yields permutations of the population indices (cf. Figure 2). Finally, in formulas, rotation and mirroring equivariance of collisions reads

10 $$\begin{aligned} \varOmega (\sigma f_i^{pre}) = \sigma \varOmega ( f_i^{pre}),\ \forall \sigma \in D_8. \end{aligned}$$

**P3**: *Mass and momentum invariance.* In the D2Q9 LBM model, mass and momentum are preserved “exactly” by the collision. This holds thanks to the underlying Gaussian quadrature used in the discretization of the velocity space [48, 49]:
  11 $$\begin{aligned} \sum _{i=0}^{8} \left( f_i^{\textrm{post}} - f_i^{\textrm{pre}} \right)&= 0, \nonumber \\ \sum _{i=0}^{8} \left( f_i^{\textrm{post}} - f_i^{\textrm{pre}} \right) \varvec{\xi }_i&= \varvec{0}. \end{aligned}$$

Finally, we shall require *positivity* (**P4**) for the post-collision lattice populations ($f_i^{post} > 0$ for all *i*), since they represent discrete velocity distribution functions.

## Machine learning approach

In this section we describe a machine learning approach, hinged on a neural network, to approximate the collision operator. Therefore, such a neural network will act as a replacement of the right hand side of Eq. 1. Our learning problem aims at finding a neural network $\varOmega ^{\textrm{NN}}$ such that $\varOmega ^{\textrm{NN}}\approx \varOmega$, i.e., formally,

12 $$\begin{aligned} {\left\{ \begin{array}{ll} \tilde{f}_i^{\textrm{post}} = \varOmega ^{\textrm{NN}} (f_i^{\textrm{pre}}), \ \ i = 0, \dots , 8,\\ \tilde{f}_i^{\textrm{post}}\approx f_i^{\textrm{post}} , \end{array}\right. } \end{aligned}$$

where the input of the network, $f_i^{\textrm{pre}}$, is given by the pre-collision (post-streaming) lattice populations, and the network output, $\tilde{f}_i^{\textrm{post}}$, targeting the post-collision populations $f_i^{\textrm{post}}$.

In the reminder of the section we will define:

- The loss function whose minimization drives the NN training process. This will also formalize our desired approximation $\tilde{f}_i^{\textrm{post}}\approx f_i^{\textrm{post}}$.
- The training and testing datasets.
- The network architecture, addressing the strategies that we considered to embed symmetries and conservations.

*Loss function and training procedure.* We train the neural network to minimize the Mean Squared Relative Error (MSRE) between ground-truth post-collision populations, $f_i^{\textrm{post}}$, and the neural network approximations, $\tilde{f}_i^{\textrm{post}}$, accumulated across the populations:

13 $$\begin{aligned} \textrm{MSRE} = \sum _{i=0}^{8} \left( \frac{\tilde{f}_{i}^{\textrm{post}} - f_{i}^{\textrm{post}}}{f_{i}^{\textrm{post}}}\right) ^2. \end{aligned}$$

Here, the use of a relative error metric is crucial in order to achieve good accuracy, since in general the lattice populations take values proportional to the corresponding lattice weights $w_i$, and, as a consequence, an absolute error metric would lead to the NN learning with higher accuracy the rest-population $f_0$ (typically the one taking the largest value) at the expense of the others.

From an implementation perspective, we consider a mini-batch stochastic gradient descent approach driven by standard adaptive moment estimation (ADAM) optimizer [50].

*Training and testing datasets* In order to control the distribution of the macroscopic parameters appearing in the training set, we rely on synthetic data rather than actual simulation data. The training set consists of *N* pairs of 9-tuples

14 $$\begin{aligned} \{( f_{i,k}^{\textrm{pre}}, \varOmega (f_{i,k}^{\textrm{pre}}) ), k=1,2,\ldots ,N\}, \end{aligned}$$

where the pre-collision distributions are generated as

15 $$\begin{aligned} f_i^{\textrm{pre}} = f_i^{\textrm{eq}}(\rho , \varvec{u}) + f_i^{\textrm{neq}} . \end{aligned}$$

In the above, the equilibrium distribution $f_i^{\textrm{eq}}$ is calculated using Eq. 3 from a set of randomly sampled macroscopic variables $\rho , \varvec{u}$. The non equilibrium part $f_i^{\textrm{neq}}$ is such that each population is randomly drawn from a Gaussian distribution, after which corrections are introduced to ensure no contributions to lower order moments, i.e.,

16 $$\begin{aligned} \sum _{i=0}^{8} f_i^{\textrm{neq}}&= 0, \nonumber \\ \sum _{i=0}^{8} f_i^{\textrm{neq}} \varvec{\xi }_i&= \varvec{0}. \end{aligned}$$

See Appendix A for further details.

**Table 1:** List of hyper-parameters used in the training of the NNs presented in this work

Number of hidden layers	2
Neurons per hidden layer	50
Hidden layer activation	ReLU
Loss function	MSRE (Eq. 13)
Optimizer	ADAM
Training dataset size	$10^{6}$
Batch size	32
Number of epochs	200
Initial learning rate	$10^{-3}$

### Neural network architectures

We consider variations of a fully connected feed-forward Neural Network, henceforth referred to as *NN Naive*, which is composed of two hidden layers of 50 neurons each. We use ReLU (rectified linear unit) as activation functions and no biases in the linear layers.

The Naive NN, as it concatenates bias-less linear layers and ReLU activations, all degree-1 homogenous functions, is itself degree-1 homogeneous. Therefore it is hardwired to respect the scale equivariance P1. Yet, no other properties such as conservation of mass, momentum and $D_8$ equivariance are imposed, thus the denomination naive.

To amend this lack, in the reminder of this section we consider three further architectures:

- *NN Sym*, satisfying properties P1, P2, P4;
- *NN Cons*, satisfying properties P1, P3;
- *NN Sym+Cons*, satisfying properties P1, P2, P3.

Before detailing the structure of these networks, we present a more general approach to satisfy P1, which we will use in all next three architectures. It hinges on considering pre- and post-collision populations normalized by the corresponding macroscopic density (invariant, P3). In formulas, we effectively consider and train a NN, $\hat{\varOmega }^{NN}$, operating as

17 $$\begin{aligned} \tilde{\phi }_i^{\textrm{post}} =\hat{\varOmega }^{\textrm{NN}}(\phi _i^{\textrm{pre}}) , \end{aligned}$$

where the normalized pre-collision populations are defined as

18 $$\begin{aligned} \phi _i^{\textrm{pre}} = f_i^{\textrm{pre}} / \rho = f_i^{\textrm{pre}} / \sum _{i=0}^8 f_i^{\textrm{pre}}. \end{aligned}$$

The normalized post-collision populations are defined analogously.

Our final collision approximator, $\varOmega ^{\textrm{NN}}$, prepends and appends rescaling operations as

19 $$\begin{aligned} \tilde{f}_i^{\textrm{post}} = \varOmega ^{NN}(f^{\textrm{pre}}_i) = \rho \hat{\varOmega }^{NN}(\phi _i^{\textrm{pre}}). \end{aligned}$$

On this basis, we can enforce positivity, P4, by considering a softmax activation function at the final layer of the network (i.e., in place of a ReLU activation). Let $y_0,\ldots ,y_8$ be the 9 inputs of the final activation, then the softmax outputs read

20 $$\begin{aligned} \tilde{\phi }_i^{\textrm{post}} = \frac{e^{y_i}}{Z}=\frac{e^{y_i}}{\sum _{i=0}^8 e^{y_i}} \ . \end{aligned}$$

Note that this returns normalized populations by construction (cf. Equation 18).

### $D_8$ equivariance: *NN Sym*

We establish a collision NN, $\bar{\varOmega }^{\textrm{NN}}$, in which we enforce the rotation and symmetry equivariance (cf. Equation 10). We achieve this by applying a $D_8$ group averaging operation on a generic collision $\varOmega ^{\textrm{NN}}$. In formulas, $\bar{\varOmega }^{\textrm{NN}}$ operates as follows:

21 $$\begin{aligned} \tilde{f}_i^{\textrm{post}} = \bar{\varOmega }^{\textrm{NN}}(f_i^{\textrm{pre}}) = \frac{1}{|D_8|} \sum _{\sigma \in D_8}\sigma ^{-1}\varOmega ^{\textrm{NN}} (\sigma f_i^{\textrm{pre}}). \end{aligned}$$

A proof that Eq. 21 satisfies P2 (Eq. 10) is provided in Appendix D. Note that this approach is general: given any symmetry group the average in Eq. 21 generates an operator that is equivariant with respect to such a group action. Note that here we perform a convex combination of populations, hence ensuring positivity of populations, with combined weight of unity, which ensures preservation of density (assuming the original operator $\varOmega ^{\textrm{NN}}$ had these properties).

In Fig. 2, we report our implementation of Eq. 21. Both at training time and for predictions the core network $\varOmega ^{\textrm{NN}}$ is evaluated 8 times on rotated/shifted versions of the input ($\sigma f_i^{\textrm{pre}}$). The outputs are then averaged after an application of the inverse rotation/shift ($\sigma ^{-1}$).

### Conservation of mass and momentum: *NN Cons*

A possible approach to ensure that Eq. 11 is satisfied, is algebraically correcting the lattice populations which the NN outputs (see also Ref [51] for an example where hard-constraints on conservation laws are imposed on the full Boltzmann equation). The method is based on the observation that all the conserved quantities are linear combinations of the lattice populations. Let

22 $$\begin{aligned} \varvec{f} = [f_0,\dots ,f_8]^T \end{aligned}$$

be the vector of the lattice populations, and $\varvec{C}$ be an invertible matrix (representing change in bases):

23 $$\begin{aligned} \varvec{C} = [\varvec{c}_0,\dots ,\varvec{c}_8]^T \end{aligned}$$

with

24 $$\begin{aligned} \varvec{c}_0 \cdot \varvec{f}&= \rho \nonumber \\ \varvec{c}_1 \cdot \varvec{f}&= u_x \nonumber \\ \varvec{c}_2 \cdot \varvec{f}&= u_y . \end{aligned}$$

Consequently, the remaining column vectors $\varvec{c}_3,\dots ,\varvec{c}_8$ are linearly independent and complementing $\varvec{c}_0,\varvec{c}_1,\varvec{c}_2$ to a base of ${\mathbb {R}}^9$.

The matrix $\varvec{C}$ represents an invertible map ${\mathbb {R}}^9\rightarrow {\mathbb {R}}^9$ which can be used to express a change in basis:

25 $$\begin{aligned} \varvec{b} = \varvec{C} \varvec{f} \Longleftrightarrow \varvec{f} = \varvec{C}^{-1} \varvec{b}. \end{aligned}$$

Thus, the first three entries of $\varvec{b}$ are the density and the momentum components.

Let $\varvec{I}_1$ and $\varvec{I}_2$ be two diagonal matrices adding up to identity matrix (i.e., $\varvec{I}_1 + \varvec{I}_2 = \varvec{I}$), and satisfying

26 $$\begin{aligned} \varvec{I}_1&= \text {diag}(1, 1, 1, 0, \dots , 0) \nonumber \\ \varvec{I}_2&= \text {diag}(0, 0, 0, 1, \dots , 1) \ . \end{aligned}$$

We define the algebraic corrections as

27 $$\begin{aligned}&\tilde{\varvec{f}}^{\textrm{post}} =\varOmega _c(\varvec{f}^{\textrm{pre}}) =\varvec{A} \varvec{f}^{\textrm{pre}} + \varvec{B} \varOmega ^{\textrm{NN}} (\varvec{f}^{\textrm{pre}}), \nonumber \\&\quad \text {with} \quad \varvec{A}=\varvec{C}^{-1}\varvec{I}_1\varvec{C} \quad \text {and} \quad \varvec{B}=\varvec{C}^{-1}\varvec{I}_2\varvec{C}. \end{aligned}$$

The choice of $\varvec{A}$ and $\varvec{B}$ is not unique. In what follows we will report results where the algebraic reconstruction is applied to the populations of index 2, 5 and 8, using:

$$\begin{aligned} \varvec{A}&= \left[ \begin{array}{rrrrrrrrr} 0 &{} 0 &{} 0 &{} 0 &{} 0 &{} 0 &{} 0 &{} 0 &{} 0\\ 0 &{} 0 &{} 0 &{} 0 &{} 0 &{} 0 &{} 0 &{} 0 &{} 0 \\ 1 &{} 0 &{} 1 &{} 2 &{} 1 &{} 0 &{} 2 &{} 2 &{} 0 \\ 0 &{} 0 &{} 0 &{} 0 &{} 0 &{} 0 &{} 0 &{} 0 &{} 0\\ 0 &{} 0 &{} 0 &{} 0 &{} 0 &{} 0 &{} 0 &{} 0 &{} 0\\ -\frac{1}{2} &{} \frac{1}{2} &{} 0 &{} -\frac{3}{2} &{} -1 &{} 1 &{} -1 &{} -2 &{} 0 \\ 0 &{} 0 &{} 0 &{} 0 &{} 0 &{} 0 &{} 0 &{} 0 &{} 0\\ 0 &{} 0 &{} 0 &{} 0 &{} 0 &{} 0 &{} 0 &{} 0 &{} 0 \\ \frac{1}{2} &{} \frac{1}{2} &{} 0 &{} \frac{1}{2} &{} 1 &{} 0 &{} 0 &{} 1 &{} 1 \\ \end{array}\right] ,\\ \varvec{B}&= \left[ \begin{array}{rrrrrrrrr} 1 &{} 0 &{} 0 &{} 0 &{} 0 &{} 0 &{} 0 &{} 0 &{} 0\\ 0 &{} 1 &{} 0 &{} 0 &{} 0 &{} 0 &{} 0 &{} 0 &{} 0 \\ -1 &{} 0 &{} 0 &{}-2 &{}-1 &{} 0 &{}-2 &{}-2 &{} 0 \\ 0 &{} 0 &{} 0 &{} 1 &{} 0 &{} 0 &{} 0 &{} 0 &{} 0 \\ 0 &{} 0 &{} 0 &{} 0 &{} 1 &{} 0 &{} 0 &{} 0 &{} 0 \\ \frac{1}{2} &{} -\frac{1}{2} &{} 0 &{} \frac{3}{2} &{} 1 &{} 0 &{} 1 &{} 2 &{} 0 \\ 0 &{} 0 &{} 0 &{} 0 &{} 0 &{} 0 &{} 1 &{} 0 &{} 0 \\ 0 &{} 0 &{} 0 &{} 0 &{} 0 &{} 0 &{} 0 &{} 1 &{} 0 \\ -\frac{1}{2} &{} -\frac{1}{2} &{} 0 &{} -\frac{1}{2} &{} - 1 &{} 0 &{} 0 &{} -1 &{} 0 \end{array}\right] . \end{aligned}$$

A second example is provided in Appendix C. Since the reconstruction occurs after the last hidden layer of the NN in general it does not ensure strictly positive lattice populations, even when used in combination with the softmax activation function (nevertheless, we never observed negative populations in the numerical results reported in the coming sections).

Note that this approach allows to enforce the conservation of mass and momentum at training time and yields no additional hyperparameters to be tuned.

An alternative approach, commonly adopted in the literature  [52–54], consists of introducing a soft constraint in the loss function in order to penalize mass and momentum mismatches. In formulas, this reads:

28 $$\begin{aligned} {\mathcal {L}} = \text {MSRE} + \alpha _1 | \tilde{\rho } - \rho | + \alpha _2 \Vert \tilde{\varvec{u}} - \varvec{u} \Vert \ , \end{aligned}$$

where $\tilde{\rho }$ and $\tilde{\varvec{u}}$ are the macroscopic quantities calculated over the lattice populations output of the network $\tilde{f_i}^{\textrm{post}}$, while $\alpha _1$ and $\alpha _2$ weights the relative importance of each single constraint.

Since we have observed that the imposition of hard constraints via algebraic reconstruction systematically outperforms the soft-constraint based approach, the latter will not be covered in our analysis in the coming sections. Nevertheless, a few numerical results are reported in Appendix B where we highlight the shortcomings of this approach.

## Numerical results

In this section, we present the results of LBM simulations where the collision term is replaced by either of the four neural networks introduced in the previous section: NN Naive, NN Sym, NN Cons, NN Sym+Cons. For each NN architecture we trained 50 instances, adopting random weights initialization. We stop the training process at 200 epochs. See Table. 1 for the full list of training hyper-parameters.

**Fig. 3:**
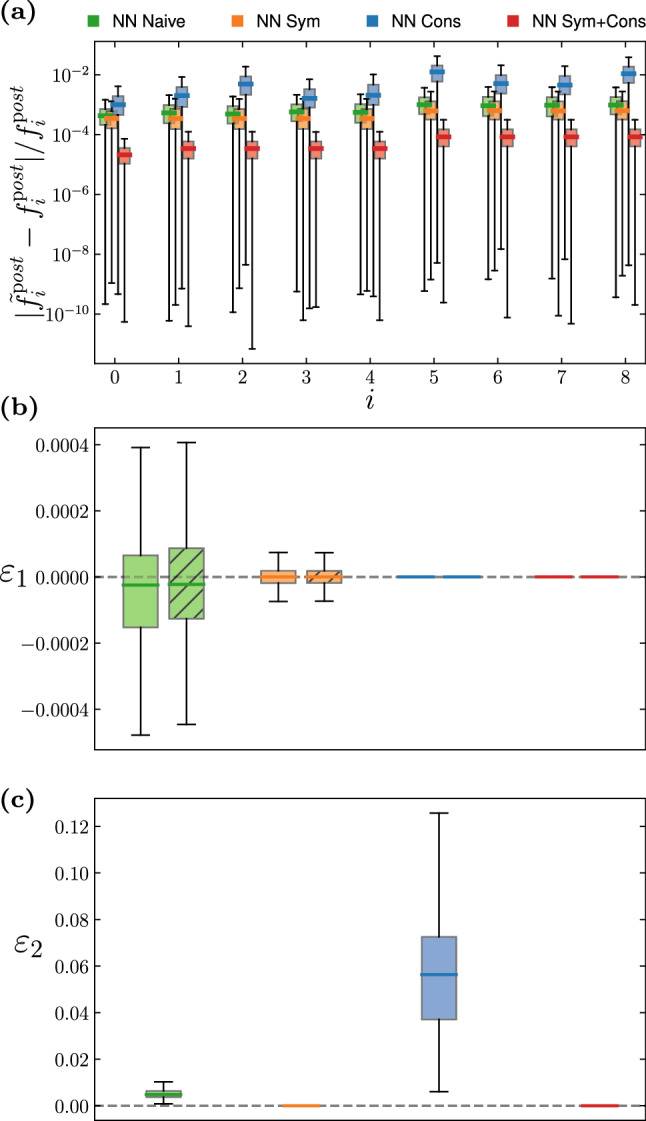
Static evaluation of the accuracy achieved by the four different NN architecture considered in this work. **a** Comparison of the absolute relative error on the post-collision populations of index *i* (cf. Figure 1). **b** Error committed in the conservation of momentum, with the uniformly filled boxplots representing the error associated with $u_x$, and the boxplots with patterned filling the error associated with $u_y$ (see Eq. 29 for the definition of the error metric). Note that the errors for NN Cons and NN Sym+Cons are zero to machine precision. **c** Error committed in violating rotation and mirroring equivariance (see Eq. 30 for the definition of the error metric). Note that for NN Sym and NN Sym+Cons the error is zero down to machine precision

We will first provide a static evaluation of the NN prediction error on the post-collision lattice populations. We also report on the physical properties of the learned collision operator. We will then turn our analysis to the comparison of time dependent flows considering two standard benchmarks: a Taylor–Green vortex decay, and a lid-driven cavity flow.

### Static accuracy evaluation

We start by comparing the accuracy of the various NN architectures described in the previous section taking into consideration the training error. In Fig. 3a we show the distribution of the absolute relative error on the post-collision populations committed by the NN on the test dataset (generated following the procedure described in Appendix A). The boxplots compare the accuracy of 50 different instances of each NN architecture in the prediction of populations of index *i*. By comparing the median values we observe that NN implementing symmetries slightly, although systematically, outperform the Naive NN. On the other hand, hardwiring conservation laws do not lead to an improvement in the prediction of the lattice populations. This is due to the specific choice of algebraically reconstructing populations of index 2, 5 and 8 to restore the conservation of mass and momentum, and it can indeed be seen from the plot that the largest errors area associated with these three elements. A major improvement is achieved when combining conservation with rotation and symmetry equivariance (*NN Sym+Cons*). This case allows to improve accuracy in the prediction of the single lattice populations between 1 and 2 order of magnitudes with respect to all the previous cases.

**Fig. 4:**
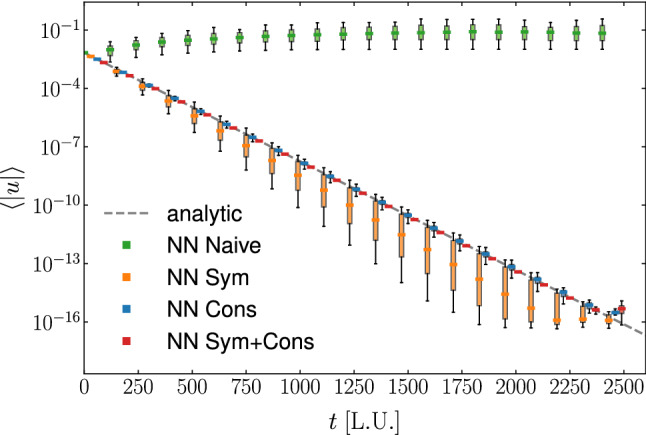
Time evolution of the average absolute value of the velocity field in a Taylor–Green vortex, comparing the analytic solution (gray dotted line) against simulations making use of NNs with different architectures. The boxplots show variability among 50 different instances for each different NN architecture. The NN with built-in symmetries and conservation properties (red) is the most accurate, followed by NN with only conservation properties (blue), followed by NN with only symmetries (orange). The naive NN (green) is the least accurate

**Fig. 5:**
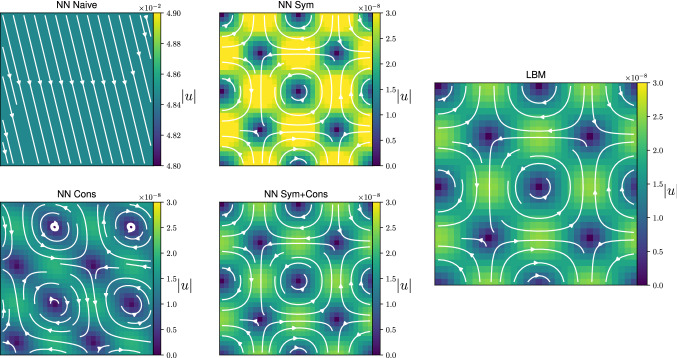
Velocity profile from simulations of a Taylor–Green vortex decay, after 1000 time steps. Color map indicates the absolute value of the velocity vector, whereas white lines provide the velocity streamlines. We compare the ground truth from a LBM simulation against the results provided by different NN implementations

We now evaluate how well the different architecture comply to the physical properties of the collision operator. In Fig. 3b we evaluate the distribution of the error committed in the momentum conservation by the various NN. We define

29 $$\begin{aligned} \varepsilon _1 = (u_j^{\textrm{pre}} - u_j^{\textrm{post}}) / c_s \ , \end{aligned}$$

with $u_j^{\textrm{pre}}$ the momentum calculated on the pre-collision distribution functions, and $u_j^{\textrm{post}}$ the momentum calculated from the distribution functions predicted by the NN; in the plot the case $j = x$ is represented by the boxplots with uniform filling, and $j = y$ by the boxplots with patterned filling.

The $\varepsilon _1$ error distribution for the Naive NN is different when comparing the two spatial components, and also asymmetric with respect to zero. We observe that the NN implementing the symmetries of the lattice (*NN Sym*) outperforms the Naive NN, in turn restoring the symmetry in the error distribution. By construction, the error for the NN implementing conservation laws is systematically zero to machine precision.

Finally, in Fig. 3c we evaluate the distribution of the following error metric

30 $$\begin{aligned} \varepsilon _2 = \frac{1}{|D_8|}\sum _{i=0}^8 \sum _{\sigma \in D_8} \Big |\sigma \varOmega (f_i^{\textrm{pre}}) -\varOmega ( \sigma f_i^{\textrm{pre}} )\Big | \ , \end{aligned}$$

which quantifies the violation of the $D_8$ equivariance. For $D_8$-equivariant collisions, i.e., satisfying P2 (Eq. 10), the term within the absolute value is zero to machine precision. We evaluate $\varepsilon _2$ over the entire test dataset. We observe that the network implementing conservation laws (*NN Cons*) commits a larger error even when comparing with the Naive NN. This is due to the fact that the algebraic reconstruction procedure used to implement the conservation laws leads to the error accumulating along some lattice directions. The error metric is systematically zero for all the NN implementing the group-averaging technique.

In the coming sections we compare the performance of the different NN in the simulation of time-dependent fluid flows.

### Benchmark I: Taylor–Green vortex

We consider the time dynamics of a Taylor–Green vortex, a standard benchmark for the validation of fluid flow solvers since it provides an exact solution to the Navier–Stokes equations.

Starting from the following initial conditions in a 2D periodic domain:

31 $$\begin{aligned} u_x(x,y)&= u_0 \cos {\left( x\right) } \sin {\left( y\right) },\nonumber \\ u_y(x,y)&=-u_0 \cos {\left( y\right) } \sin {\left( x\right) }, \quad x,y \in [0, 2 \pi ] \end{aligned}$$

with $u_0$ the initial value for $|\varvec{u}|$, it is simple to show that the flow decays exponentially and proportionally to

32 $$\begin{aligned} F(t) = \exp {\left( -2 \nu t \right) } , \end{aligned}$$

where $\nu$ is the kinematic viscosity of the fluid (Eq. 5). This benchmark allows us to evaluate the time dynamic of a flow, covering different orders of magnitude in the values of the macroscopic parameters, and also to evaluate the preservation of symmetries by observing the structure of the vortexes.

We consider a $32 \times 32$ grid, with $u_0 = 10^{-2}$, $\tau = 1$. In Fig. 4, we compare the time decay of the average absolute value of the velocity field from simulations making use of different NNs, comparing against the analytic solution. Once again, for each type of NN we have evaluated the results from 50 different networks trained starting from a random choice of the initial weights. The plot highlights the variability in the results from the different NNs by means of boxplots. From the plot we can see that the Naive NN is able of correctly follow the flow decay for only 20-40 iterations, after which not only the flow stops decaying but we also observe an increase in the kinetic energy. By employing a NN satisfying the symmetries of the lattice it is possible to restore the decaying trend of the flow, although we observe a deviation from the correct decaying rate. This can be attributed to the network not being able of preserving momentum. On the other hand, NNs enforcing the conservation laws are able to provide a more accurate dynamic, with only small variability around the analytic solution, which can be further reduced by combining conservation and preservation of symmetries.

**Fig. 6:**
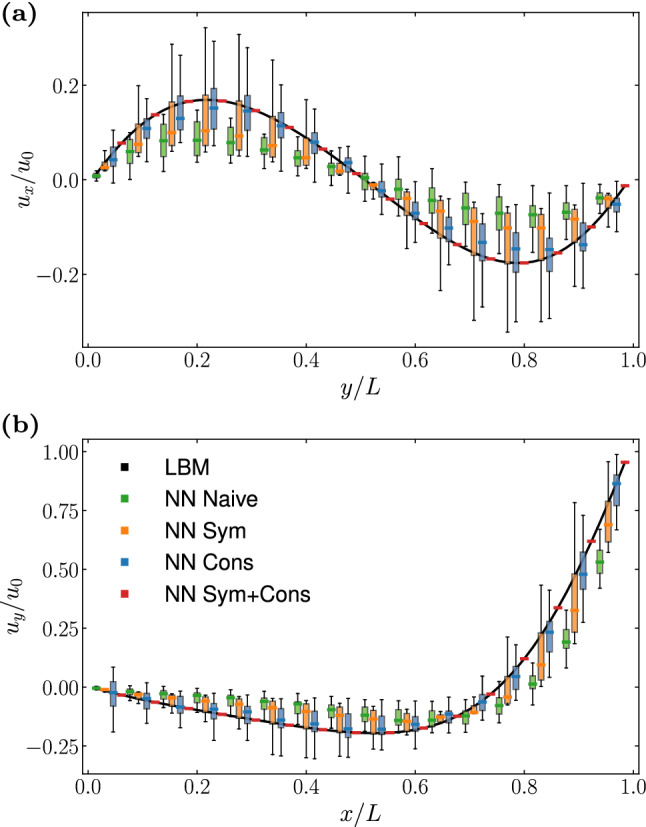
Steady state profiles for **a** $u_x$ along the vertical centerline, and **b** $u_y$ along the horizontal centerline of the domain of a lid-driven cavity flow at $\textrm{Re} = 10$. Simulations are performed on a square grid of side $L = 64$. We compare the results of a LBM simulation (black line), against results obtained employing the four NN architectures considered in this work. The boxplots report the variability in the results from 50 instances of each NN architecture

**Fig. 7:**
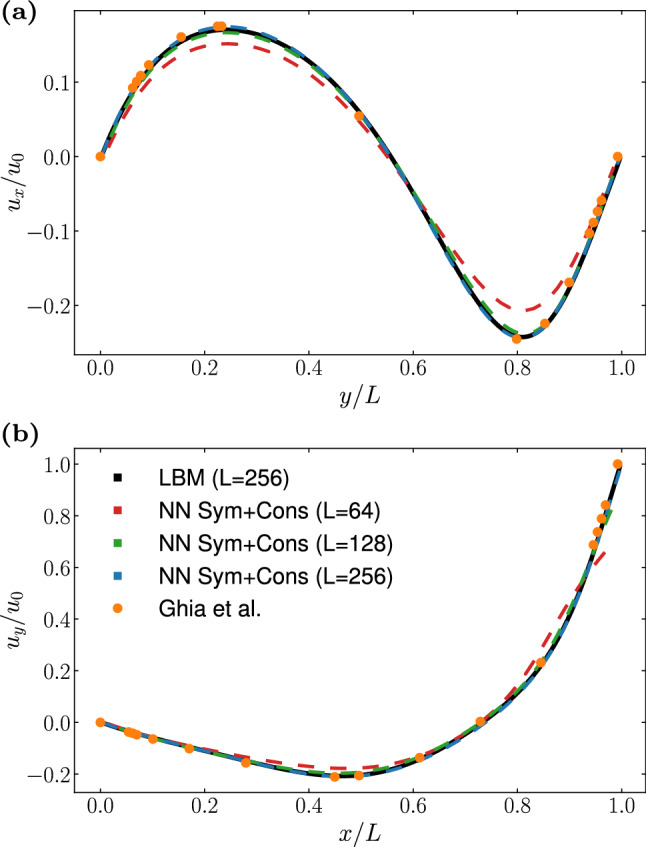
Steady state profiles for **a** $u_x$ along the vertical centerline, and **b** $u_y$ along the horizontal centerline of the domain of a lid-driven cavity flow at $\textrm{Re} = 100$. Dotted lines represent results obtained using a *NN Sym+Cons* architecture for increasing number of nodes in the grid side *L*. We compare the results against a LBM simulation (black line, $L = 256$), and reference data from Ghia et al. [55] (orange dots, $L = 129$)

The importance of embedding conservation laws and symmetries together in the NN is even more evident in Fig. 4, where evolution statistics is shown for four types of NN designs. Embedding symmetries or conservation properties shows an immediate and dramatic improvement over the naive NN in the ability of the NN to capture the decay rate of the average velocity field. Enforcing conservation properties is appreciably more important (for the purpose of this test) than enforcing symmetries. Yet, enforcing both symmetries and conservation properties produces the most accurate results capturing the decay of average velocity with minimal variability all the way to machine precision, which is a remarkable result, especially compared to the performance of a naive NN. Moreover, we should stress that a NN with a lower training error will not necessarily guarantee for better results when employed in simulations; for example, NN Cons, which in Fig. 3a presents the larger training error, is among the best performing one when looking at Fig. 4.

On a more qualitative basis, in Fig. 5 we provide snapshots of the velocity field at a later stage of the dynamics (after $t = 1000$ iterations), comparing the ground truth given by a plain LBM simulation against an example of the profile provided by each of the different NN implementations. The figure shows that, besides failing to reproduce the decay of the flow, the Naive NN is also not able to preserve the structure of the vortexes. The NN with symmetries, on the other hand, nicely preserves the geometric structure, although the amplitude of the velocity is slightly off with respect to the reference LBM profile. The NN enforcing conservation laws correctly capture on average the decaying rate (c.f. Figure 4), however, Fig. 5 clearly shows that the structure of the vortexes is not symmetric anymore. This can be attributed to the fact that the algebraic reconstruction is performed on 3 lattice populations, leading to a less balanced distribution of the error (cf. Figure 3c). Only by combining conservation and symmetries in the NN it is possible to reproduce correctly the velocity profile.

### Benchmark II: Lid driven cavity flow

As a second example, we consider the lid-driven cavity flow, a wall-bounded benchmark in a very simple geometry, still leading to a non-trivial dynamic. Indeed there is no analytic solution for this flow, and for this reason we will compare this time only against reference LBM simulations.

The setup consists of a top-lid moving at a constant velocity ($u_0$), with no-slip boundary conditions at bottom and side walls. We consider a $L \times L$ grid, the relaxation time set to $\tau = 1$, and report the results for simulations at two different Reynolds numbers, respectively, $\textrm{Re} = 10$ and $\textrm{Re} = 100$, with

33 $$\begin{aligned} \textrm{Re} = \frac{u_0 L}{\nu } \ . \end{aligned}$$

**Fig. 8:**
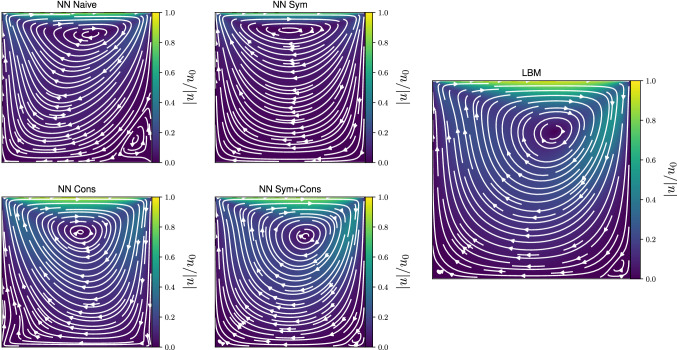
Steady state profile of the velocity field for a lid-driven cavity flow at $\textrm{Re} = 100$, comparing the results of a LBM simulation against the results provided by different NN implementations. Colors map the absolute value of the fluid velocity normalized over the lid velocity, whereas white lines provide the velocity streamlines. Simulations are performed on a square grid of side $L = 128$

In simulations the NN does not handle the evolution of the boundary nodes. Instead, we employ standard LBM approaches for implementing the boundary conditions. In particular, the bounce back rule is used to implement the no-slip condition. Here the lattice populations that during the streaming step interact with a solid wall get reflected to their original location with their velocity reversed:

34 $$\begin{aligned} f_{\bar{i}} (\varvec{x}, t + 1) = f_i (\varvec{x}, t) \ , \end{aligned}$$

where $f_{\bar{i}}$ is the population of index $\bar{i}$ such that $\varvec{\xi }_{\bar{i}} = - \varvec{\xi }$. For the top wall we use a Dirichlet boundary condition

35 $$\begin{aligned} f_{\bar{i}} (\varvec{x}, t + 1) =f_i (\varvec{x}, t) + 2 w_i \rho _w \frac{\varvec{\xi }_i \cdot \varvec{u}_w}{c_s^2} \ , \end{aligned}$$

where $\rho _w$ and $\varvec{u}_{w} = (u_0, 0)$ are respectively the density and the velocity at the top wall.

In Fig. 6, we show the steady state velocity profiles along the vertical (a) and horizontal (b) centerlines of the lid-driven cavity for $\textrm{Re} = 10$, comparing the results from a plain LBM simulation against results obtained employing NNs with different architectures. All simulations are performed on a square grid of side $L = 64$. Once again we show data collected simulating 50 different instances of each NN architecture, with the boxplots reporting the variability in the obtained results. We observe that in this case the results of the Naive NN are much closer to the reference data with respect to the previous benchmark. This can be attributed to the boundary conditions constraining the flow. Both *NN Sym* and *NN Cons* provide an improvement over the Naive NN, however it is interesting to point out that the results provided by the latter present a much higher variability than the one observed in the simulation of the Taylor–Green vortex. Indeed, the plot clearly shows that only the case *NN Sym+Cons* is able to correctly reproduce the results of the LBM simulation. We select this NN architecture to perform simulation at a higher Reynolds number. In Fig. 7 we show the results obtained at $\textrm{Re} = 100$, varying the grid size, and comparing with both a LBM simulation as well as with reference data from Ghia et al. [55]. The results from the simulation using the finer grid resolution ($L=256$) are found to be in excellent agreement with the reference data. On the other hand, we see that for coarser grid sizes the NN struggles to correctly reproduce the velocity in the proximity of the moving plate (see Fig. 7b). We shall discuss the origin of this mismatch in the coming subsection.

In Fig. 8, we show a more qualitative comparison for the case $\textrm{Re} = 100$, presenting snapshots of the velocity field at the steady state, and comparing the results from a LBM simulation with results produced by the different NN architectures. It is interesting to observe that each different NN make a different prediction for the location of the main vortex, and only few reproduce the secondary vortex located at the bottom right corner. As expected from the analysis above, *NN Sym+Cons* provides results in excellent agreement with the LBM simulation.

### Extrapolation

**Fig. 9:**
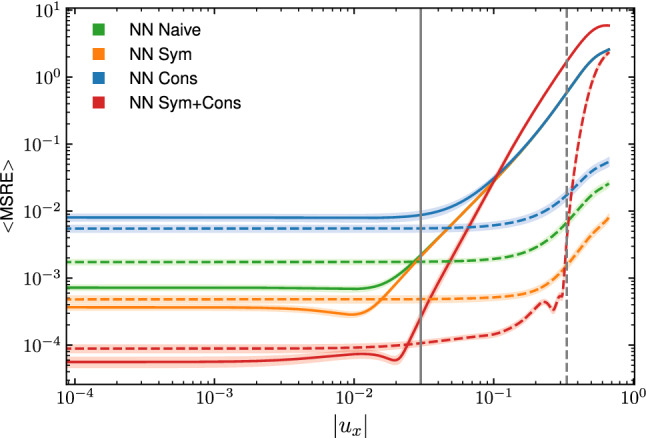
Comparison for the accuracy of the different NNs architecture within and outside the training dataset. The plot shows the average MSRE, computed on 50 instances of each NN architecture, in the prediction of the equilibrium distribution $f_i^{\textrm{eq}}(\rho = 1, u_x, u_y=0)$ at varying values of $u_x$. The continuous lines refer to NNs trained on a dataset where the macroscopic velocity takes values in the interval $(-0.03, 0.03)$, while the interval $(-1/3, 1/3)$ has been used to train the NNs corresponding to the dotted lines. The gray continuous (dotted) vertical lines identify the boundary of the two training datasets

In Fig. 7b, we have observed significant deviations in the numerical results produced by the *NN Sym+Cons* architecture in the proximity of the moving plate, in particular for coarse grids. Since in simulations we are keeping fixed the kinematic viscosity and the Reynolds number, it follows from Eq. 33 that by increasing the grid resolution we also decrease the numerical value of the lid velocity $u_0$. For $L=64$ the numerical value used at the top lid $u_0 \approx 0.26$ falls well outside the range of values shown to the network at training time. It is, therefore, interesting to investigate the extrapolation capabilities of the different NNs. In Fig. 9, we show the average MSRE on 50 instances of each NN architecture, calculated in the prediction of the equilibrium distribution $f_i^{\textrm{eq}}(\rho = 1, u_x, u_y=0)$ at varying values of $u_x$. The continuous lines show the performance of the NNs trained on a dataset where the macroscopic velocity takes values in the interval $(-0.03, 0.03)$; likewise, the dotted lines show the results for NNs trained on values of the macroscopic velocity in the interval $(-1/3, 1/3)$. Corresponding gray continuous (dotted) vertical lines are reported to identify the boundary of the two training datasets. Here we can see that when working in the range of values shown to the NN during the training, the *NN Sym+Cons* outperforms all the other network architectures. On the other hand, this NN commits the largest extrapolation error, i.e., it commits a larger error in predicting the equilibrium distribution outside of the values of the training set. While the reason for this behavior is currently unclear to us and will be object of further analysis in future work, these results explain the discrepancies observed in Fig. 7, where simulations with numerical values for the top-lid, which were outside of the training dataset, led to larger discrepancies with respect to the reference solution. This, in turn, points to the need of extra care in the preparation of the training dataset.

## Conclusion

In this work, we have presented a machine learning approach for learning a collision operator for the Lattice Boltzmann Method from data. As a proof of concept, we have developed a neural network capable of approximating to good accuracy the BGK collision operator. We have discussed in details a few methods which allow enriching the structure of the neural network to enforce relevant physical properties of the collision operator. We have shown that only by embedding conservation laws and lattice symmetries in the neural network it is possible to correctly reproduce the time dynamics of a fluid flow.

This work can be regarded as a first step toward the application of neural networks for extending the applicability of LBM in kinematic regimes not supported by the basic method. To give an example, in future extensions of the present work, we plan to evaluate the possibility of using our approach for learning collision operators from molecular dynamics and Monte Carlo simulations in regimes beyond hydrodynamic limit. While moving in this direction we expect that dealing with boundary conditions will become increasingly important, and one can think of training multiple NN for implementing diverse type of boundary conditions.

Moreover, we will take into consideration other approaches for embedding symmetries in the network to allow for a scalable extension to the 3-dimensional case, and for employing higher order stencils.

## Figures and Tables

**Fig. 10:**
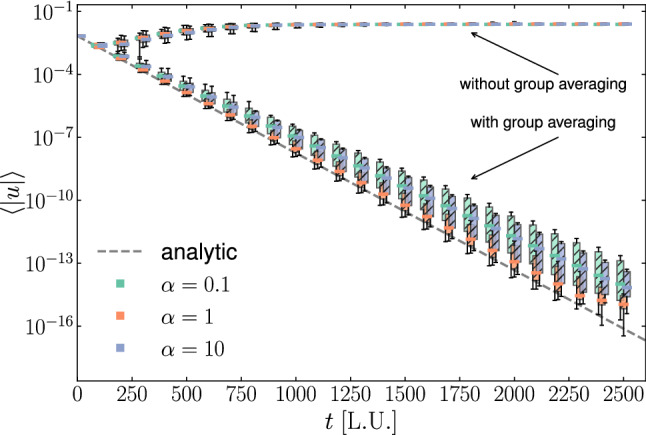
Time evolution of the average absolute value of the velocity field in a Taylor–Green vortex, comparing the analytic solution (gray dotted line) against simulations making use of NNs employing a soft-constraint on momentum conservation, for a few selected values of the parameter $\alpha$ which weights the relative importance of the soft-constraint (see Eq. 44). For each value of $\alpha$ we show results obtained with and without the group-averaging method used for embedding symmetries in the NN architecture. The boxplots show variability among 20 different instances for each different NN architecture

## Data Availability

This manuscript has associated data in a data repository. [Authors’ comment: A minimal set of scripts, based on Keras and Tensorflow, allowing to (i) generate the training dataset (ii) train a neural network and (iii) plug the neural network in a LBM simulation, can be found at https://github.com/agabbana/learning_lbm_collision_operator.

## Appendix A: Training data generation algorithm

In this appendix section, we summarize the steps followed in the generation of the training dataset. While the procedure described in Algorithm 1 is general, we provide values which are specific for the D2Q9 model (for example the coefficients in Eq. 40).

## Appendix B: Conservation with soft-constraints

In Sect. 3, we have discussed the possibility of employing soft-constraints in order to impose conservation of mass and momentum. In this appendix, we report the results obtained training a NN with the following architecture: (i) same hyperpameters as in Table 1, (ii) softmax activation function at the final layer, combined with the rescaling operations in Eq. 19, (iii) an additional term in the loss function penalizing the violation of momentum conservation:

44 $$\begin{aligned} {\mathcal {L}} = \text {MSRE} + \alpha \frac{ \Vert \tilde{\varvec{u}} - \varvec{u} \Vert }{ \Vert 1 + \varvec{u} \Vert } , \end{aligned}$$

where $\tilde{\varvec{u}}$ is the velocity vector computed over the lattice populations output of the NN, and $\alpha$ is a parameter which weights the importance of the soft constraint. Note that mass conservation is already ensured by the combination of the softmax activation function with the rescaling of input and output.

We have scanned several values of the parameter $\alpha$, for which we report here three representative cases: $\alpha = 0.1, 1, 10$. For each of these selected values of $\alpha$ we have trained 20 NNs. In Fig. 10, we present the results obtained on the Taylor Green vortex benchmark described in the main text. For the case where no symmetries are enforced in the NN, the results are inline with those reported for the Naive NN in Fig. 4, i.e., we do not correctly reproduce the decaying behavior if not for very few time steps. By repeating the training embedding symmetries in the NN architecture (Fig. 2), results improve significantly. From the plot we can observe that by tuning $\alpha$ it is possible to adjust the variability in the results produced by the different instances of the NN. Still, we not in general achieve the correct decaying rate.

These results show that NNs imposing conservation laws via hard constraints systematically outperforms the soft-constraints based approach, with the added advantage of not requiring tuning of extra parameters (such as $\alpha$ in the example above).

## Appendix C: Symmetric algebraic reconstruction

In the main text, we have described one possible way to apply algebraic reconstruction to hardwire conservation laws in the NN. In particular we have considered an example which involves adjusting 3 of the 9 populations outputted by the NN. This approach, which may introduce a slight bias along those lattice directions, was useful to expose the relative importance of embedding different physical properties in the NN architecture.

However, as mentioned in Sect. 3, there are several possible approach for imposing conservation of mass and momentum in the NN.

A more “symmetric” approach, which we found to give excellent results even when not combined with the group-averaging method for embedding symmetries in the NN, reads as follows:

45 $$\begin{aligned} \tilde{f_i}^{\textrm{post}} =\varOmega ^{\textrm{NN}}(f_i^{\textrm{pre}}) + \kappa _1 + \kappa _2 \xi _{i, x} + \kappa _3 \xi _{i, y} \end{aligned}$$

where the parameters $\kappa _1, \kappa _2, \kappa _3$ are lattice dependent, and for the D2Q9 are given by:

46 $$\begin{aligned} \kappa _1&= - \frac{1}{9} \sum _{i=0}^8 \left( \varOmega ^{\textrm{NN}}(f_i^{\textrm{pre}}) - f_i^{\textrm{pre}} \right) \nonumber \\ \kappa _2&= - \frac{1}{6} \sum _{i=0}^8 \left( \varOmega ^{\textrm{NN}}(f_i^{\textrm{pre}}) - f_i^{\textrm{pre}} \right) \xi _{i, x} \nonumber \\ \kappa _3&= - \frac{1}{6} \sum _{i=0}^8 \left( \varOmega ^{\textrm{NN}}(f_i^{\textrm{pre}}) - f_i^{\textrm{pre}} \right) \xi _{i, y} \end{aligned}$$

## Appendix D: The group-averaged operator $\bar{\varOmega }$ satisfies $D_8$ equivariance

We prove here that the the group averaged operator defined in Eq. 21, respects property P2 (Eq. 10). The proof that we propose here is indeed general and holds for any symmetry group. For this reason, we indicate here the symmetry group with the generic symbol ${\mathcal {S}}$.

### Proof

We shall show that

$$\begin{aligned} \bar{\varOmega }(\eta f) = \eta \bar{\varOmega }(f), \quad \forall \eta \in {\mathcal {S}}. \end{aligned}$$

By definition, it holds

$$\begin{aligned} \bar{\varOmega }(\eta f)&= \frac{1}{|{\mathcal {S}}|}\sum _{\sigma \in {\mathcal {S}}} \sigma ^{-1} \varOmega ^{\textrm{NN}}(\sigma \eta f) \\&= \frac{1}{|{\mathcal {S}}|}\sum _{\sigma \in {\mathcal {S}}}\eta \eta ^{-1} \sigma ^{-1} \varOmega ^{\textrm{NN}}(\sigma \eta f) \\&= \eta \frac{1}{|{\mathcal {S}}|}\sum _{\sigma \in {\mathcal {S}}}(\sigma \eta )^{-1} \varOmega ^{\textrm{NN}}(\sigma \eta f) \\&= \eta \frac{1}{|{\mathcal {S}}|}\sum _{\sigma \in {\mathcal {S}}\eta }\sigma ^{-1} \varOmega ^{\textrm{NN}}(\sigma f). \end{aligned}$$

Yet, ${\mathcal {S}}\eta = {\mathcal {S}}$ or, equivalently, the presence of $\eta$ yields a permutation of the terms to add (by uniqueness of the inverse within a group). Thus, we can write

$$\begin{aligned} \bar{\varOmega }(\eta f)&= \eta \frac{1}{|{\mathcal {S}}|}\sum _{\sigma \in {\mathcal {S}}} \sigma ^{-1} \varOmega ^{\textrm{NN}}(\sigma f) \\&= \eta \bar{\varOmega }( f), \end{aligned}$$

which concludes the proof. $\square$

## References

[1] Shan X, Chen H (1993). Phys. Rev. E.

[2] Sbragaglia M, Benzi R, Biferale L, Succi S, Sugiyama K, Toschi F (2007). Phys. Rev. E.

[3] Chen H, Kandasamy S, Orszag S, Shock R, Succi S, Yakhot V (2003). Science.

[4] Philippi PC, Hegele LA, dos Santos LOE, Surmas R (2006). Phys. Rev. E.

[5] Scagliarini A, Biferale L, Sbragaglia M, Sugiyama K, Toschi F (2010). Phys. Fluids.

[6] Aharonov E, Rothman DH (1993). Geophys. Res. Lett..

[7] Gabbanelli S, Drazer G, Koplik J (2005). Phys. Rev. E.

[8] Asinari P, Mishra SC, Borchiellini R (2010). Numer. Heat Transf. Part B: Fundam..

[9] R.C. Coelho, M.M. Doria, Comput. Fluids **165**, 144–159 (2018). ISSN 0045-7930 10.1016/j.compfluid.2018.01.019

[10] A. Gabbana, D. Simeoni, S. Succi, R. Tripiccione, Phys. Rep. **863**, 1–63 (2020). 10.1016/j.physrep.2020.03.004

[11] S. Succi, The Lattice Boltzmann Equation: For Complex States of Flowing Matter. OUP Oxford (2018). ISBN 9780192538857. 10.1093/oso/9780199592357.001.0001

[12] S. Succi, G. Amati, M. Bernaschi, G. Falcucci, M. Lauricella, A. Montessori, Comput. Fluids **181**, 107–115 (2019). 10.1016/j.compfluid.2019.01.005

[13] Bhatnagar PL, Gross EP, Krook M (1954). Phys. Rev..

[14] I. Ginzburg, F. Verhaeghe, D. d’Humières, Commun. Comput. Phys. **3** 427–478 (2008). https://hal.inrae.fr/hal-02589582

[15] D’Humières D (1992). Progress Astronaut. Aeronaut..

[16] Lallemand P, Luo LS (2000). Phys. Rev. E.

[17] J. Latt, B. Chopard, Math. Comput. Simul. **72** 165–168 (2006). 10.1016/j.matcom.2006.05.017

[18] Zhang R, Shan X, Chen H (2006). Phys. Rev. E.

[19] Mattila KK, Philippi PC, Hegele LA (2017). Phys. Fluids.

[20] Karlin IV, Gorban AN, Succi S, Boffi V (1998). Phys. Rev. Lett..

[21] Ansumali S, Karlin IV (2002). Phys. Rev. E.

[22] Meng J, Zhang Y, Hadjiconstantinou NG, Radtke GA, Shan X (2013). J. Fluid Mech..

[23] Ambruş VE, Sofonea V (2018). Phys. Rev. E.

[24] Latt J, Coreixas C, Beny J, Parmigiani A (2020). Philos. Trans. R. Soc. A: Math. Phys. Eng. Sci..

[25] Coreixas C, Chopard B, Latt J (2019). Phys. Rev. E.

[26] K. Hornik, M. Stinchcombe, H. White, Neural Networks **2**, 359–366 (1989).10.1016/0893-6080(89)90020-8

[27] N. Sebe, I. Cohen, A. Garg, T.S. Huang, *Machine learning in computer vision*, vol. 29 (Springer, Berlin, 2005). 10.1007/1-4020-3275-7

[28] J. Wieting, M. Bansal, K. Gimpel, K. Livescu (2015). arXiv preprint arXiv:1511.08198

[29] R. King, O. Hennigh, A. Mohan, M. Chertkov, (2018). arXiv preprint arXiv:1810.07785

[30] Karniadakis GE, Kevrekidis IG, Lu L, Perdikaris P, Wang S, Yang L (2021). Nat. Rev. Phys..

[31] Ling J, Kurzawski A, Templeton J (2016). J. Fluid Mech..

[32] Tian Y, Livescu D, Chertkov M (2021). Phys. Rev. Fluids.

[33] Wang JX, Wu JL, Xiao H (2017). Phys. Rev. Fluids.

[34] A.T. Mohan, N. Lubbers, D. Livescu, M. Chertkov (2020). arXiv preprint arXiv:2002.00021

[35] Duraisamy K, Iaccarino G, Xiao H (2019). Ann. Rev. Fluid Mech..

[36] Ortali G, Corbetta A, Rozza G, Toschi F (2022). Phys. Rev. Fluids.

[37] Mohan AT, Tretiak D, Chertkov M, Livescu D (2020). J. Turbulence.

[38] Fukami K, Fukagata K, Taira K (2019). J. Fluid Mech..

[39] G.D. Portwood, P.P. Mitra, M.D. Ribeiro, T.M. Nguyen, B.T. Nadiga, J.A. Saenz, M. Chertkov, A. Garg, A. Anandkumar, A. Dengel et al. (2019). arXiv preprint arXiv:1911.05180

[40] Corbetta A, Menkovski V, Benzi R, Toschi F (2021). Sci. Adv..

[41] Beintema G, Corbetta A, Biferale L, Toschi F (2020). J. Turbulence.

[42] O. Hennigh, (2017). arXiv preprint arXiv:1705.09036

[43] X. Guo, W. Li, F. Iorio, Convolutional neural networks for steady flow approximation. Proceedings of the 22nd ACM SIGKDD International Conference on Knowledge Discovery and Data Mining (New York, NY, USA: Association for Computing Machinery) pp 481–490 (2016). ISBN 9781450342322 10.1145/2939672.2939738

[44] Wang YD, Chung T, Armstrong RT, Mostaghimi P (2021). Transp. Por. Med..

[45] M.C. Bedrunka, D. Wilde, M. Kliemank, D. Reith, H. Foysi, A. Krämer, *Lettuce: Pytorch-based lattice boltzmann framework* (Springer, Cham, 2021), pp.40–55. 10.1007/978-3-030-90539-2_3

[46] Krüger T, Kusumaatmaja H, Kuzmin A, Shardt O, Silva G, Viggen EM (2017). The Lattice Boltzmann Method.

[47] Chapman S, Cowling TG (1970). The Mathematical Theory of Non-Uniform Gases.

[48] Shan X (2010). Phys. Rev. E.

[49] Shan X (2016). J. Comput. Sci..

[50] D.P. Kingma, J. Ba (2014). arXiv preprint arXiv:1412.6980

[51] S.T. Miller, N.V. Roberts, S.D. Bond, E.C. Cyr, J. Comput. Phys. **470** 111541 (2022). ISSN 0021-9991 10.1016/j.jcp.2022.111541

[52] S.L. Brunton, Acta Mechanica Sinica 1–9 (2022). 10.1007/s10409-021-01143-6

[53] A. Dener, M.A. Miller, R.M. Churchill, T. Munson, C.S. Chang (2020). arXiv preprint arXiv:2009.07330

[54] Kim B, Azevedo VC, Thuerey N, Kim T, Gross M, Solenthaler B (2019). Comput. Gr. Forum.

[55] U. Ghia, K. Ghia, C. Shin, J. Comput. Phys. **48** 387–411 (1982). 10.1016/0021-9991(82)90058-4

